# Exploring urinary microbiome: insights into neurogenic bladder and improving management of urinary tract infections

**DOI:** 10.3389/fcimb.2025.1512891

**Published:** 2025-04-01

**Authors:** Jinming Zhang, Yingyun Lei, Huayong Du, Zehui Li, Xiaoxin Wang, Degang Yang, Feng Gao, Jianjun Li

**Affiliations:** ^1^ School of Rehabilitation Medicine, Capital Medical University, Beijing, China; ^2^ Department of Spinal and Neural Functional Reconstruction, China Rehabilitation Research Center, Beijing, China

**Keywords:** microbiome, urine, neurogenic bladder, urinary tract infections, diagnostics, treatment

## Abstract

The traditional view of sterile urine has been challenged by advancements in next-generation sequencing, revealing that the urinary microbiome significantly influences individual health and various urinary system diseases. Urinary tract infections in patients with neurogenic bladder are highly prevalent, recurrent, and lifelong. If frequent urinary tract infections are not adequately managed, they may ultimately lead to chronic renal failure. The excessive use of antibiotics to prevent and treat urinary tract infections may lead to increased bacterial resistance, limiting future therapeutic options. This review summarizes commonly used microbiome research techniques and urine collection methods, compiles current studies on the urinary microbiome in neurogenic bladder patients, and discusses the potential implications of urinary microbiome composition for preventing, diagnosing, and treating urinary tract infections. By summarizing current research findings, we aim to enhance understanding of the urinary microbiome in neurogenic bladder patients and promote the standardization and clinical translation of microbiome research.

## 1 Introduction

**Figure 1:**
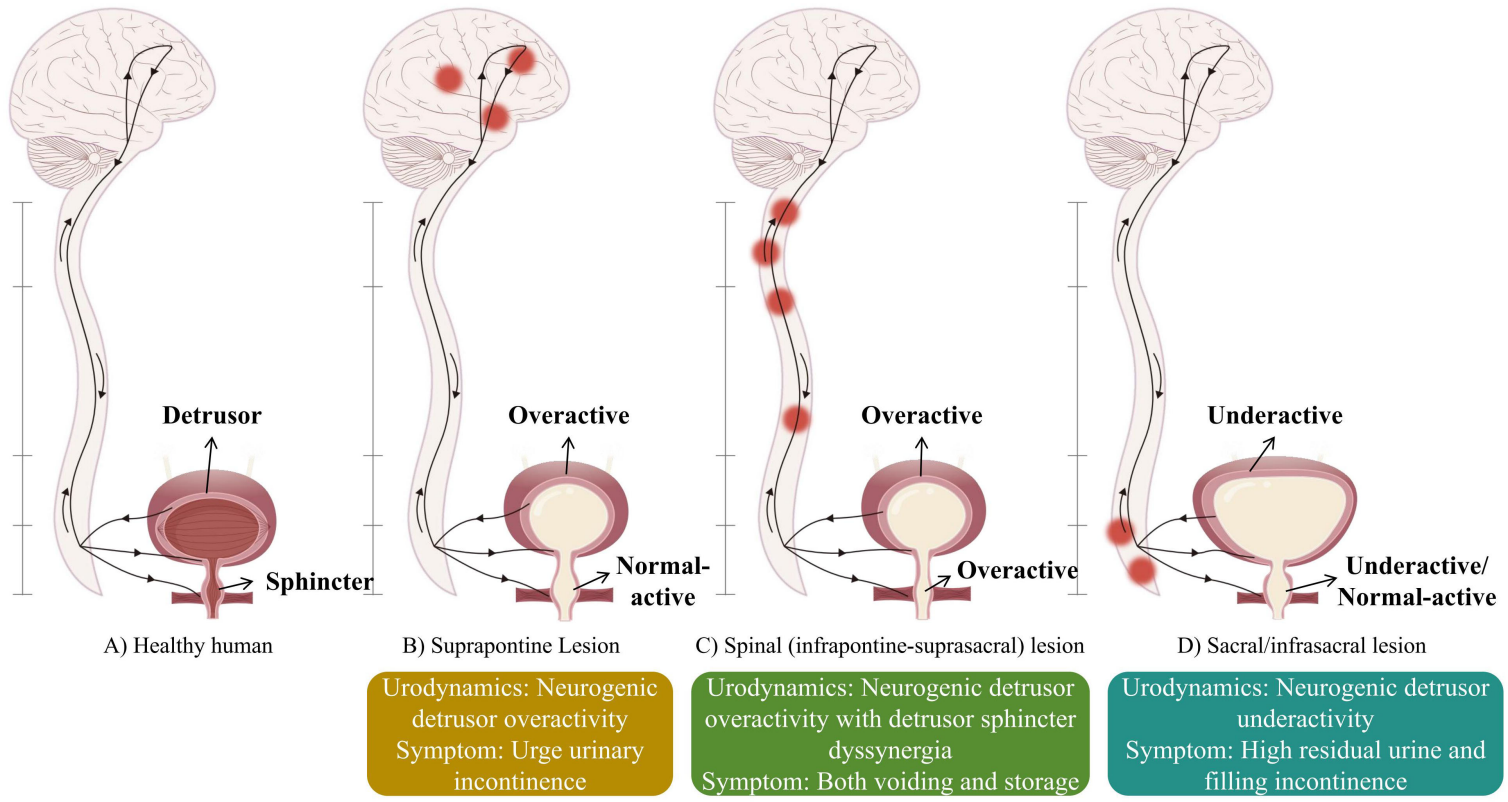
Different lesion locations lead to different types of neurogenic bladder.

Neurogenic bladder (NB) refers to a range of lower urinary tract dysfunctions caused by neurological disorders, manifesting as urinary incontinence, urinary retention, or a combination of both (Amarenco et al., 2017). It severely impacts the patient’s quality of life and causes embarrassment, discomfort, and social isolation (Alghamdi et al., 2023). In addition to the inability to control urination, NB can result in severe complications such as urinary tract infections (UTIs), urinary tract stones, vesicoureteral reflux, and autonomic dysreflexia (Ginsberg et al., 2021). The clinical presentation of NB depends on the location of the neurological lesion and its relationship with the central nervous system (**Figure 1**) (Kumar and Biswas, 2022). The most common causes of NB include spinal cord injury (SCI), multiple sclerosis, Parkinson’s disease, and cerebrovascular disease (Panicker, 2020).

Lower urinary tract dysfunction is the core manifestation of NB and an important trigger for recurrent urinary tract infections (rUTIs). Chronic ischemia of the bladder wall caused by lower urinary tract pressure, bladder mucosal damage caused by overdistension, and frequent catheterization collectively constitute the most challenging clinical complication of rUTIs (Linsenmeyer, 2018). NB Patients have their urinary systems constantly exposed to a high-risk environment for bacterial colonization, significantly increasing the occurrence rate of rUTIs (Milicevic et al., 2024). Recurrent infections exacerbate underlying symptoms such as frequency, urgency, and incontinence, further damaging the patient’s quality of life. The continuous invasion by pathogens and the synergistic effect of high bladder pressure increases the risk of upper urinary tract injury. Studies indicate that the prevalence of chronic kidney disease in NB patients is more than three times higher than in the general population, and chronic renal failure is often the outcome of this pathological process (Kari, 2006; Sung et al., 2018).

Treatment strategies for NB include conservative approaches (fluid management, pelvic floor muscle exercises, clean intermittent catheterization, and indwelling catheters), pharmacological treatments (anticholinergic drugs and alpha blockers), non-pharmacological interventions (sacral nerve stimulation and acupuncture), and surgical options (botulinum toxin injections, augmentation cystoplasty, and continent cutaneous urinary diversion) (DeWitt-Foy and Elliott, 2022). In recent years, significant advancements have been made in the management of NB and the prevention of its complications (Koukourikis et al., 2023), such as the use of mesenchymal stem cells to improve impaired bladder function and the application of urinary interleukin-6 to distinguish asymptomatic bacteriuria (ASB) from non-febrile UTIs (Sundén et al., 2017; Chen et al., 2022).

Despite substantial progress in the diagnostic methods and therapeutic strategies for NB and its urological complications, there remains an unmet clinical need for early diagnostic tools and novel effective treatments for UTIs in NB patients. Research into the human microbiome has provided valuable perspectives on the microbial composition of the urinary system and its changes under pathological conditions, potentially aiding in the development of novel prevention, diagnostic, and treatment strategies. Studies on gut microbiota have demonstrated that microbial communities and their metabolites interact with the human immune system and various diseases, such as inflammatory bowel disease, irritable bowel syndrome, colorectal cancer, and neurodegenerative disorders (Sebastián Domingo and Sánchez Sánchez, 2018; Goyal et al., 2021; Si et al., 2021). Similarly, the urinary microbiome is closely related to urinary system diseases, including UTIs, overactive bladder, bladder cancer, interstitial cystitis, etc (Lee et al., 2020).

This review summarizes commonly used techniques for urinary microbiome research and key considerations for sample collection. We also review current studies on the urinary microbiome in healthy individuals and NB patients and analyze the microbiome’s dynamic changes in different situations. Furthermore, we explore the microbiome’s potential as a distinct biomarker for early prediction and diagnosis, along with its role in rUTIs prevention and treatment.

## 2 Literature search and selection criteria

We systematically searched for literature published before 2024 to assess the current research progress on the urinary microbiome in neurogenic bladder and its application in urinary tract infections. We searched PubMed, Web of Science, and Google Scholar databases, focusing on high-quality relevant studies. The included studies focused on the urinary microbiome composition in healthy individuals and NB patients, its mechanisms in UTIs, and therapeutic interventions like probiotic therapy. Articles that were not peer-reviewed, case reports, unpublished abstracts, and non-English studies were excluded. A total of 28 studies met the predefined inclusion criteria after screening. Keywords used in the search included “healthy,” “neurogenic bladder,” “neurogenic lower urinary tract dysfunction,” “urine,” “urinary,” “microbiome,” “microbiota,” “fungi,” “virus,” “virome,” “phage,” “metagenome,” “urinary tract infection,” “probiotics,” “fecal microbiota transplantation,” and “therapy.”

## 3 Microbiome research methods

Traditionally, urinary microbiome detection has relied on standard urine cultures. However, this method has low detection rates for slow-growing and anaerobic microorganisms (Legaria et al., 2022). Next-generation sequencing (NGS) has facilitated the comprehensive characterization of the urinary microbiome, with primary methods including 16S rRNA gene sequencing (16S rRNA GS) and whole genome shotgun sequencing (WGS) (Ishihara et al., 2020).

Researchers extract DNA from samples using DNA isolation kits such as PowerSoil and BiOstic (Karstens et al., 2021), and then subsequently choose whether to perform sequencing via 16S rRNA GS or WGS. The 16S rRNA gene is universally present in all bacteria and archaea, with a full length of approximately 1500 bp, containing 9 variable regions (V1-V9) and 10 conserved regions (Abellan-Schneyder et al., 2021). Most 16S rRNA GS protocols use the V3-V4 or V4 hypervariable regions for PCR amplification and sequencing, which can classify most microorganisms to the genus level. Third-generation sequencing platforms, such as PacBio SMRT, can provide full-length sequencing to identify species-level diversity in certain microorganisms (Buetas et al., 2024). WGS involves randomly fragmenting microbial genomic DNA, adding universal primers to the ends of the fragments for PCR amplification and sequencing, and then assembling the short fragments into longer sequences. This method enables the detection of all microorganisms (including fungi and viruses) and provides their complete genomic sequences (Zhou et al., 2022).

Both 16S rRNA GS and WGS have their advantages and disadvantages. 16S rRNA GS primarily investigates microorganisms’ types, relative abundance, and evolutionary relationships. With second-generation sequencing technology, 16S rRNA GS typically only identifies microorganisms at the genus level without accurately determining species or subspecies levels and does not provide genomic sequence information beyond the primer-specified amplicon. WGS, on the other hand, allows for in-depth analysis of species classification, gene function, and metabolic networks based on 16S rRNA GS. However, it is more costly, and researchers need to choose the appropriate method based on their research objectives.

After selecting the method, techniques such as Illumina MiSeq can be used for adapter ligation, PCR amplification, library preparation, and sequencing. The resulting sequences can be analyzed using computational tools like QIIME2, Kraken2, and MetaPhlAn3 to obtain sequencing results (Min et al., 2022).

A limitation of microbial sequencing is its inability to distinguish live from dead bacteria (Aragón et al., 2018). Enhanced quantitative urine culture (EQUC), an improvement of the standard urine culture (SUC) protocol, has been developed to address this. SUC involves inoculating 1 μL of urine onto blood agar or MacConkey agar plates and incubating under standard ambient atmospheric, aerobic conditions at 35°C for 18-24 hours. In contrast to SUC, EQUC has a lower detection threshold (10 CFU/mL, compared to the 1000 CFU/mL of SUC), more diverse culture conditions (blood agar plate, MacConkey, chocolate, and colistin-nalidixic acid agars), growth temperatures (20°C, 30°C, 35°C), atmospheric conditions (air, CO_2_, anaerobic), incubation times (24-48 hours), and urine volumes (1, 10, and 100 μL) (Gasiorek et al., 2019). EQUC expands the range of potentially detectable live bacteria, including aerobic, microaerophilic, slow-growing anaerobic, facultative anaerobes, and fastidious bacteria (Deen et al., 2023). In urinary microbiome research, EQUC has shown higher sensitivity than SUC in detecting urogenital pathogens (Price et al., 2016; Modena et al., 2017).

Since microbial community classification analysis has been the primary objective in most urinary microbiome studies, the convenient and cost-effective 16S rRNA GS method has been the predominant approach (Neugent et al., 2020). Increasingly, researchers are combining 16S rRNA GS with EQUC or WGS to enhance detection and analysis (Antunes-Lopes et al., 2020).

## 4 Urine sample collection

Determining the adequate volume of urine to obtain sufficient bacterial DNA is crucial, as the microbial biomass in urine is relatively low, averaging less than 10^4^ CFU/ml (O’Hara and Shanahan, 2006). Based on current studies involving the urinary microbiome, collecting at least 10 ml of urine is generally considered feasible (Xu et al., 2021; Retchless et al., 2022; Yoo et al., 2022).

Contamination is another challenge in urine collection. The primary methods include midstream voiding, catheterization, and suprapubic aspiration. Due to the proximity of the urethral opening to other high-microbe-density anatomical structures, such as the urothelium, surrounding glands, vagina, or intestines, bacterial contamination can introduce sampling bias (Kim et al., 2022). Significant differences in *Lactobacillus*, *Neisseria*, and *Veillonella* are observed between midstream urine samples and catheterized samples (Pohl et al., 2020; Hrbacek et al., 2021). Although suprapubic aspiration avoids contact with other areas and provides a more accurate microbiome composition of urine (Eliacik et al., 2016), it is complex and less well-accepted by patients. Existing studies suggest that the microbiome composition in urine obtained via suprapubic aspiration and catheterization is essentially similar. Hence catheter-based urine sample collection is widely employed in urinary microbiome research (Wolfe et al., 2012).

Finally, since urine cannot always be analyzed immediately after collection, appropriate storage methods are crucial for the accuracy and reproducibility of research. Studies vary in terms of urine storage time, temperature, and the use of DNA preservatives (Karstens et al., 2016; Komesu et al., 2017). Typically, urine should be transferred to 4°C within 4 hours of collection and then frozen at -80°C for long-term storage. Preservatives like AssayAssure^®^ do not introduce significant microbial bias, and it is recommended to add them directly at a 1:10 ratio (Brubaker et al., 2021a). Research indicates that using preservatives, minimizing storage time, and maintaining lower temperatures are conducive factors for preserving the integrity of the urinary microbiome (Jung et al., 2019). In cases where neither preservatives nor temporary refrigeration is available, the urine should be promptly transferred to -80°C within 3 hours after collection.

## 5 Urinary microbiome composition in healthy individuals

To understand the characteristics of the urinary microbiome in NB patients, the first step is to identify which microbiome members are consistently found in the urine of healthy individuals. The urinary microbiome in healthy populations exhibits individual variations, with its composition influenced by ethnicity, age, sex, and diet. In general, the urinary microbiome demonstrates a gender—and age-dependent composition (**Table 1**).

**Table 1:** Urinary microbiome of healthy individuals.

Author	Enriched Genera	Urine collection	Detection method
Storm et al.	MC: *Streptococcus* (Only in 3-12 years) FC: *Bifidobacterium*, *Veillonella* (<3 years). *Veillonella, Prevotella*, *Dialister, Haemophilus*, and *Schaalia* (3-12 years). *Lactobacillus*, *Bifidobacterium*, *Gardnerella* (12+ years).	Catheterization	EQUC and 16S rRNA GS
Wehedy et al.	MC: *Anaerococcus*, *Xylanimonas*, *Arthrobacter*, *Acidothermus* FC: *Anaerococcus*, *Veillonella*	Midstream urine from spontaneous voiding	WGS
Fouts et al.	M: *Corynebacterium* *F: Lactobacillus*	Midstream urine from spontaneous voiding	SUC and 16S rRNA GS
Pohl et al.	M: *Streptococcus* *F: Lactobacillus*	Midstream urine from spontaneous voiding and catheterization	SUC and 16S rRNA GS
Price et al.	F: *Lactobacillus*, *Streptococcus*, *Gardnerella*, *Escherichia*	Catheterization	EQUC and 16S rRNA GS
Modena et al.	M: *Streptococcus*, *Lactobacillus*, *Prevotella*, *Corynebacterium*, *Pseudomonas* F: *Lactobacillus*, *Corynebacterium*, *Gardnerella*, *Prevotella*, *Corynebacterium*	Not mentioned	16S rRNA GS
Curtiss et al.	F: *Staphylococcus*, *Streptococcus*, *Lactobacillus*	Midstream urine from spontaneous voiding	16S rRNA GS

MC, male children; FC, female children; M, male; F, female; EQUC, expanded quantitative urine culture; SUC, standard urine culture; 16S rRNA GS, 16S rRNA gene sequencing; WGS, whole genome shotgun sequencing.

At the phylum level, both male and female urinary microbiomes are dominated by Firmicutes, which make up more than two-thirds of the entire microbiome composition, followed by Actinobacteria, Bacteroidetes, and Proteobacteria (Pohl et al., 2020; Perez-Carrasco et al., 2021). At the genus level, male children (<18 years) generally lack significant characteristic microbiota, with the only notable feature being *Streptococcus* in prepuberty (3-12 years). The urinary microbiome predominantly comprises *Corynebacterium*, *Pseudomonas*, and *Prevotella* in adult males. Female urinary microbiomes exhibit changes according to developmental stages, with *Bifidobacterium* and *Veillonella* being the most common in the pre-toilet-trained stage (<3 years). There is higher genus diversity in prepuberty, with *Veillonella*, *Prevotella*, and *Dialister* dominating. Microbial diversity decreases post-puberty (>12 years), and *Lactobacillus* dominates, similar to the adult female microbiome. In adult females, the urinary microbiome is predominantly composed of *Lactobacillus*, *Streptococcus*, and *Gardnerella* (Fouts et al., 2012; Curtiss et al., 2017; Pohl et al., 2020; Storm et al., 2022). *Gardnerella* and *Lactobacillus* are more frequently found in younger women, whereas *Escherichia* and *Mobiluncus* are more common in postmenopausal women (Curtiss et al., 2018; Price et al., 2020a).

It is difficult to establish a unified and constant definition of the urinary microbiome in healthy populations, and these age- and sex-related variations are important considerations when evaluating the urinary microbiome and its role in health and disease. Understanding these patterns is crucial for developing targeted therapies and diagnostic tools based on urinary microbiome composition.

## 6 Urinary microbiome composition in neurogenic bladder patients

The urinary microbiome in NB patients is disrupted compared to healthy individuals, with key features including an increase in pathogenic pathogens and a decrease in the abundance of protective microbiota (**Table 2**). Existing studies indicate that the disease, bladder management methods, and disease progression influence this ecological imbalance. Fouts conducted a comparative analysis of the urinary microbiome in 27 NB patients with spinal cord injury and 26 healthy controls, and the study revealed NB patients had significantly decreased abundances of *Lactobacillus*, *Corynebacterium*, and *Prevotella* compared to healthy controls. At the same time, *Klebsiella*, *Escherichia*, and *Enterococcus* increased significantly. In female NB patients, regardless of the urination method, the relative abundance of *Lactobacillus* was lower than that in healthy female controls (Fouts et al., 2012). Further analysis showed that the abundance of *Lactobacillus* in NB patients decreased by twofold compared to healthy controls (P=0.02), with the key species *Lactobacillus crispatus* nearly disappearing, while *Escherichia coli* (P=0.005) and *Enterococcus faecalis* (P=0.028) significantly increased (Groah et al., 2016). Lane’s study highlighted that the urinary microbiome of NB patients is generally dominated by a single species, with Unclassified *Enterobacteriaceae* being the most prevalent, followed by *Escherichia* (Lane et al., 2022). This “pathogen-protection” imbalance is similarly prominent in the pediatric NB group. Forster investigated the urinary microbiome of NB children using clean intermittent catheterization (CIC) and found that the most abundant bacteria were Unspecified *Enterobacteriaceae* (mean abundance 56%), followed by *Klebsiella* (19%), *Staphylococcus* (7%), and *Streptococcus* (3%), consistent with studies in adults (Forster et al., 2020). De Maio found that the abundance of Proteobacteria (commonly considered the major urinary tract pathogens in NB patients) was significantly higher in NB children compared to healthy controls (35.5% vs. 2.8%, p<0.001). At the genus level, *Bifidobacterium*, *Enterococcus*, and *Escherichia* abundances were higher in NB children (9.2% vs. 1.6%, 10.8% vs. 1.2%, 28.6% vs. 3.8%, p<0.001).

**Table 2:** Urinary microbiome of patients with neurogenic bladder.

Author	Study population	Sample collection	Main bacterial taxa in control group	Main bacterial taxa in patients with neurogenic bladder (NB)	Technique used
Fouts et al.	27 NB Adults due to spinal cord injury and 26 healthy adults	NB patients: spontaneous voiding, intermittent catheterization, and indwelling Foley catheterization. Healthy adults: normal voiding	*Lactobacillus*, *Corynebacterium*, *Staphylococcus*, *Streptococcus*, *Prevotella*, *Veillonella*	*Klebsiella*, *Escherichia*, *Enterococcus, Aerococcus*, *Brevibacterium*, *Salmonella*, *Proteus*	SUC and 16S rRNA GS
Lane et al.	69 NB Adults due to various etiologies	Intermittent catheterization, indwelling urinary catheters, and suprapubic drainage.	/	*Escherichia*, *Proteus*, *Enterococcus*, *Pseudomonadaceae*, *Staphylococcus*, *Streptococcus*, *Aerococcus*, *Lactobacillus*, *Lachnospiraceae*, *Anaerococcus*, *Gardnerella*, *Corynebacterium*, *Prevotella*	SUC and 16S rRNA GS
Forster et al.	34 NB children aged 2 months to 21 years	Clean intermittent catheterization (via urethra or Mitrofanoff stoma)	/	Unspecified *Enterobacteriaceae*, *Klebsiella*, *Staphylococcus*, *Streptococcus, Enterococcus*, *Bacteroides*, *Gemella*, *Veillonella*, Unspecified *Neisseriaceae*, *Dialister*	16S rRNA GS
De Maio et al.	44 NB children aged 1-18 years due to spina bifida and 32 healthy controls	NB children: intermittent catheterization and spontaneous voiding Healthy controls: normal voiding	*Bacteroides*, *Ezakiella*, *Lactobacillus*, *Peptoniphilus*, *Prevotella*	*Bacteroides*, *Bifidobacterium*, *Cutibacterium*, *Enterococcus*, *Escherichia*, *Shigella*, *Lactobacillus*, *Subdoligranulum*	16S rRNA GS

NB patients often use indwelling catheters and CIC for bladder emptying. Invasive catheterization procedures may remodel the urinary microbiome structure through several mechanisms, including physical damage to the urinary epithelium, loss of key colonizing microbiota, promotion of pathogen biofilm formation, and disruption of the anatomical barrier leading to cross-ecological niche microbial displacement. Research has shown that NB patients using suprapubic drainage and CIC have significantly higher levels of *Enterobacteriaceae* and lower levels of *Lactobacillaceae* compared to non-NB patients. This difference was not observed in NB patients who had spontaneous voiding. In NB patients, those using catheters had higher urinary microbiome diversity than spontaneous voiding patients. Among them, patients using CIC had lower *α* diversity compared to those using indwelling catheters (1.16 vs 1.85, p=0.01), with no significant difference in *β* diversity (Groah et al., 2016; Lane et al., 2022). A similar conclusion was found in another study, where CIC patients exhibited higher *α* and *β* diversity in their urinary microbiomes than non-CIC patients. The abundance of Bacteroidetes in CIC samples was significantly lower than in non-CIC samples (10.7% vs 18.1%, p = 0.02), whereas Proteobacteria was more abundant in CIC samples (41.1% vs 21.2%, p = 0.02). At the genus level, *Cutibacterium* was relatively more abundant in CIC patient samples than in non-CIC samples (4.9% vs 1.2%, p = 0.01), while *Faecalibacterium*, *Lactobacillus*, *Staphylococcus*, and *Streptococcus* showed significantly lower abundances (De Maio et al., 2023).

Studies on the time dimension have revealed a trend of urinary microbiome imbalance in NB patients. The urinary microbiome in healthy individuals exhibits dynamic variability and resilience against disturbances. For example, in women, the α-diversity of the urinary microbiome increases under the influence of menstruation and sexual activity, with an increased abundance of *Streptococcus* and *Staphylococcus*. However, the microbiome quickly returns to baseline after these disturbances (Price et al., 2020b). Longitudinal studies on NB patients show a loss of the dynamic balance of the urinary microbiome compared to healthy populations. Within 2 months after injury, the microbiome composition is statistically similar to that of healthy controls, but after 1 year, *Lactobacillus* almost disappears, and *Enterococcus* and *Escherichia* become predominant (Fouts et al., 2012). This dynamic evolution suggests that after developing NB, the urinary microbiome loses its resistance to disturbances, and changes in the microbiome may contribute to the high risk of UTIs after NB.

## 7 Fungi and viruses in urine

Fungi and viruses are frequently neglected parts of urinary microbiome studies, comprising only a tiny fraction of the urine microbiome. Research using WGS shows that eukaryotes and viruses account for 0.05% and 0.0027% (Moustafa et al., 2018), most of which cannot be isolated through urine culture (Ackerman and Underhill, 2017). Research in healthy individuals indicates that the urinary microbiome commonly contains fungi such as *Saccharomyces*, *Malassezia*, *Candida*, *Clavispora*, and *Meyerozyma* (Moustafa et al., 2018; Wehedy et al., 2022), as well as viruses like HPV, BK polyomavirus, JC polyomavirus, and bacteriophages (Santiago-Rodriguez et al., 2015; Garretto et al., 2018; Moustafa et al., 2018; Garretto et al., 2019). These microbes’ abundance changes may correlate with multiple diseases. Nickel observed significantly higher detection rates of *Candida* and *Saccharomyces* during flares of urological chronic pelvic pain syndrome (15.7% vs. 3.9%) (Nickel et al., 2016), and *Candida* and *Malassezia* showed higher abundance in patients with more severe symptoms compared to those with milder symptoms (Nickel et al., 2020). Moreover, Adenovirus, HPV, and cytomegalovirus infections have been associated with lower urinary tract symptoms (Brubaker et al., 2021b). Several studies have identified the lytic activity of phages against common pathogens of UTIs and used them as an alternative therapy to antibiotics to inhibit multidrug-resistant uropathogens (Chegini et al., 2021; Subramanian, 2024).

Our understanding of fungal and viral diversity in the urinary tract of NB patients is still in its nascent stages, with a plethora of unexplored microbial communities remaining to be investigated. Documenting the differences in the microbiome between healthy individuals and symptomatic patients is a crucial initial step towards comprehending the potential role played by specific components of this class of urinary microbes in lower urinary tract health. Recent studies have observed potential associations between viral communities and overactive bladder, noting differences in the abundance of Actinobacteriophages and JC virus between asymptomatic and symptomatic individuals (Miller-Ensminger et al., 2018; Sun et al., 2022). However, there is currently a lack of targeted research and comprehensive descriptions of urinary fungi and viruses among NB patients. Their interactions with other constituents within the urinary microbiome and their associations with clinical symptoms and complications following NB remain pressing issues requiring resolution.

## 8 Translating microbiome research into clinical applications

### 8.1 Potential applications of the microbiome in diagnosing and predicting UTIs

**Figure 2:**
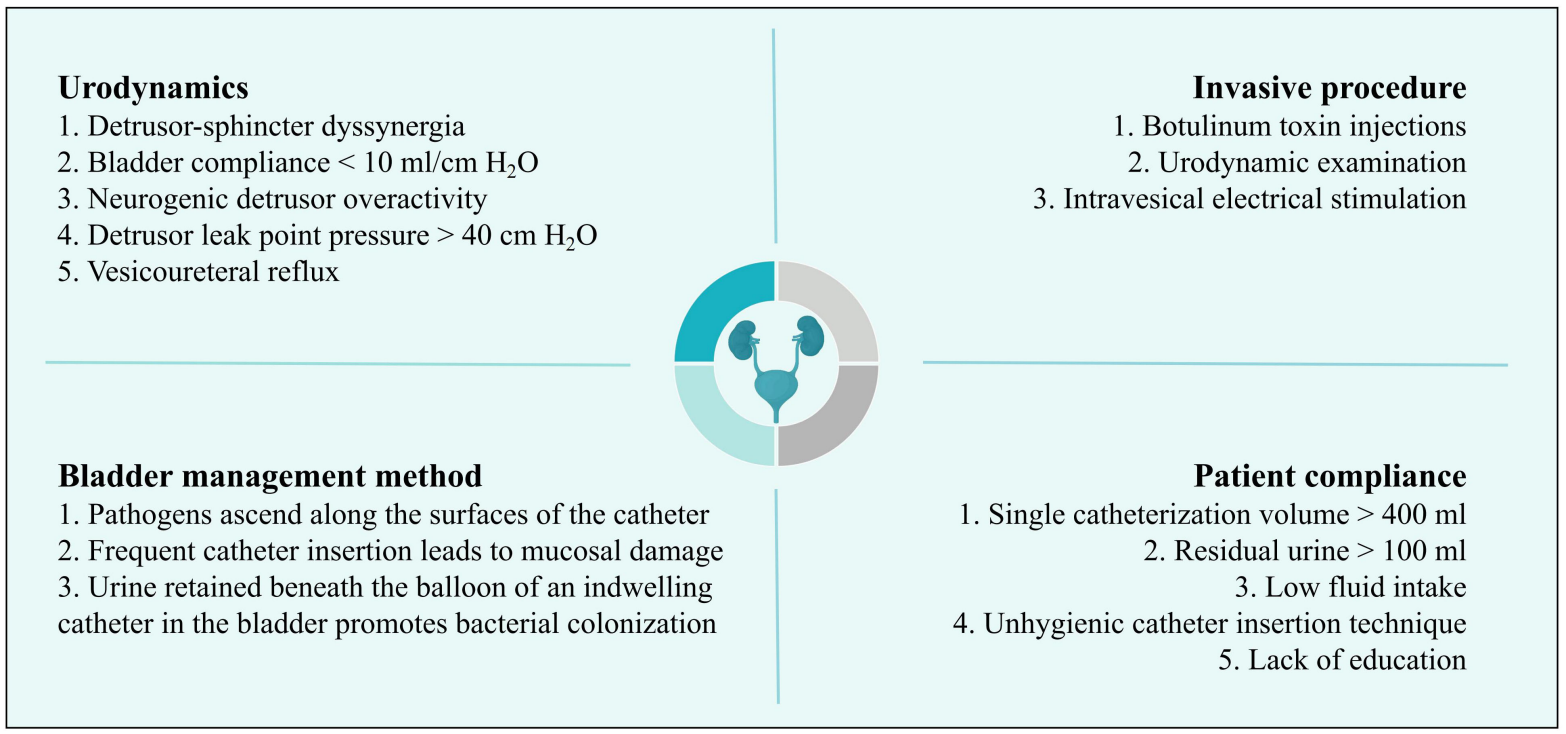
Risk factors for urinary tract infection in patients with neurogenic bladder.

Accurately distinguishing between ASB and UTIs in NB patients remains a significant challenge. ASB is defined as the presence of ≥10^5^ CFU/ml of a single uropathogen in a clean-catch midstream urine culture without clinical signs and symptoms (Luu and Albarillo, 2022), which is particularly prevalent in NB patients, with 50% to 75% of their urine cultures showing positive results. UTIs are one of the most frequent complications in patients with NB, with a prevalence of about 70% (**Figure 2**) (Penders et al., 2003; Liu et al., 2023; Fitzpatrick et al., 2024).

However, conventional UTIs diagnostic criteria—urinary symptoms (such as dysuria, odor, and cloudiness), inflammatory markers (pyuria, WBC ≥10/ml), and positive urine cultures—are often unreliable in NB patients due to the altered bladder physiology compared to health individuals (Goebel et al., 2021). The etiology of NB, NB classification, bladder management strategies, the patient’s level of voluntary sensation, and the nervous system status all influence clinical presentation and symptom reporting (Tractenberg et al., 2018). For instance, SCI patients with NB often have reduced or absent sensory perception below the injury level (Jahromi et al., 2014). Research indicates that only 61% of SCI patients with NB can accurately predict when they are amid UTIs (Linsenmeyer and Oakley, 2003).

Catheter use further complicates diagnosis. Catheterization is a common bladder management technique for NB patients; a single microbial pathogen often causes short-term catheter-associated UTIs, but most UTIs in patients with long-term catheterization (>30 days) are essentially multiple microbial infections (Klein and Hultgren, 2020). SUC are designed to detect a limited number of common aerobic urinary pathogens. This approach inevitably leads to high false negatives and diagnostic limitations of SUC in UTIs (Price et al., 2016).

There is no widely acceptable and precise definition for UTIs in NB patients. The empirical treatment approaches often lead to overdiagnosis of UTIs and unnecessary antibiotic use, which contributes to an increasing rate of antibiotic-resistant infections in these patients (Forster et al., 2017; Fitzpatrick et al., 2018). In order to improve diagnostic accuracy, reduce the recurrence of UTIs, and address the growing issue of antibiotic resistance, there is a pressing need for more precise methods explicitly tailored for NB patients. Techniques such as 16S rRNA GS, EQUC, and WGS are expected to provide more comprehensive detection and monitoring of the dynamic changes in the urinary microbiome (McDonald et al., 2017; Almas et al., 2023; Zhao et al., 2024). These methods hold promise for accurately identifying the urinary tract status at a given time and using the presence of characteristic microorganisms or specific changes in the abundance of resident microbiota as diagnostic, predictive, and prognostic markers for UTIs.

### 8.2 Emerging microbial therapies in UTIs research

UTIs is the most common complication in NB patients. RUTIs, defined as three or more episodes annually or two or more episodes within six months, account for about 30% of NB patients (Theisen et al., 2020). Frequent pathogens in rUTIs comprise *Escherichia coli*, *Klebsiella*, *Proteus*, *Pseudomonas*, and *Enterococcus*, with *Escherichia coli* responsible for over half of these infections (Dinh et al., 2020; Weyler et al., 2021; Alghoraibi et al., 2023). Although antibiotics are effective in the short term, their prolonged use to achieve the traditional “sterile urine” aim disrupts protective and beneficial urinary microbiota. Recent studies associate this with “urinary tract microecosystem dysbiosis,” which increases the risk of pathogenic colonization, escalates multidrug-resistant strains in the urinary tract, and exacerbates the challenges in managing UTIs, eventually results in upper urinary tract damage, kidney function deterioration, sepsis, and other severe consequences, significantly impacting lower urinary tract health and long-term prognosis (Finucane, 2017; Wu et al., 2022). Treatments based on the urinary microbiome offer an alternative paradigm for NB patients to combat UTIs beyond antibiotic therapies by maintaining the ecological balance of the lower urinary tract, replenishing protective characteristic taxa, or directly targeting urinary pathogens (**Table 3**).

**Table 3:** Application of probiotic therapy in urinary tract infections.

Author	Study aim	Patients	Administration	Dose&Duration	Urinary microbiome of experimental group	Urinary microbiome of control group
Beerepoot et al.	Comparison of the effectiveness of probiotics with antibiotics in preventing UTIs	253 Postmenopausal women with rUTIs	Oral	Trimethoprim-sulfamethoxazole (480 mg, qd, 12 month); *Lactobacillus rhamnosus* GR-1 and *Lactobacillus reuteri* RC-14 (10^9^ CFU per capsule, one capsule bid, 12 month)	/	/
Wolff et al.	Effects of probiotic therapy on the ratio of uropathogens to *Lactobacillus* in the lower urinary tract	7 Healthy premenopausal women	Oral	*L. rhamnosus* GR-1 and *L. reuteri* RC-14 (10^9^ CFU per capsule, one capsule bid, 40 days)	*Lactobacillus crispatus*, *Gardnerella*, *Streptococcus agalactiae*, *Staphylococcus epidermidis*, *Corynebacterium tuberculostearicum*, *Lactobacillus iners*, *Lactobacillus jensenii*	*Lactobacillus jensenii*, *Lactobacillus crispatus*, *Gardnerella*, *Streptococcus mitis*
Bossa et al.	Impact of probiotics on the urinary microbiome	3 male NB patients due to cervical SCI	Oral	*L. rhamnosus* GR-1 and *L. reuteri* RC-14 (5.4 × 10^9^ CFU per capsule, one capsule qd, 6 month); *L. rhamnosus* GG with *Bifidobacterium* BB-12 (7 × 10^9^ CFU per capsule, one capsule qd, 6 month)	*Enterobacteriaceae*, *Providencia*, *Alcaligenaceae*, *Enterococcaceae*, *Pseudomonas*, *Veillonella*	/
Toh et al.	Determine the preventive effect of RC14-GR1 and/or LGG-BB12 on UTIs in NB patients following SCI.	53 NB patients due to SCI	Oral	*L. rhamnosus* GR-1 and *L. reuteri* RC-14 (5.4 × 10^9^ CFU per capsule , one capsule qd, 6 month) + *L. rhamnosus* GG with *Bifidobacterium* BB-12 (7 × 10^9^ CFU per capsule, one capsule qd, 6 month)	Only RC14-GR1:*MRGN*↓	/
Daniel et al.	The preventive effect of probiotics therapy on UTIs in pediatric populations	54 children with rUTIs	Oral	*Lactobacillus rhamnosus* PL1 and *Lactobacillus plantarum* PM1 (10^9^ CFU per capsule, one capsule qd, 90 days)	/	/
Mula et al.	A comparison of outcomes from antibiotic treatment with and without probiotics	897 patients with UTIs	Oral	ProBalans capsules (*Lactobacillus acidophilus* LA3, *Bifidobacterium animalis lactis* BLC1, and *Lactobacillus casei* BGP93, 10^9^ CFU per capsule, one capsule bid, during antibiotic treatment and for 7 days after the completion of antibiotic therapy)	*Escherichia coli* ↓, *Staphylococcus aureus* ×, *Streptococcus agalactiae* ×, *Enterococcus* ×, *Proteus mirabilis* ×	*Escherichia coli ↓, Staphylococcus aureus* ×*, Streptococcus agalactiae ↓, Enterococcus ↓, Proteus mirabilis* ×
Stapleton et al.	The effectiveness of probiotic vaginal suppositories in preventing rUTIs	100 premenopausal women with rUTIs	Vaginal suppositories	Osel's Lactin-V (*Lactobacillus crispatus*, 10^8^ CFU/mL, one suppository qd for 5 days and then qw for 10 weeks)	/	/
Sadahira et al.	The effectiveness of vaginal suppositories in treating recurrent cystitis in postmenopausal women	21 postmenopausal women with recurrent cystitis	Vaginal suppositories	*Lactobacillus* vaginal suppositories containing *Lactobacillus crispatus* GAI 98332 (10^8^ CFU per suppository, one suppository three times a week before bed for 1 year)	/	/
Forster et al.	Determine the tolerance and safety of a single intravesical probiotic instillation	10 children with spina bifida and adults with SCI	Intravesical instillation	Culturelle (*L. rhamnosus* GG, 20 billion live organisms, once)	*Escherichia/Shigella*, *Streptococcus*, *Prevotella* ↑ *Pseudomonas* ↓ *Veillonella*, *Lactobacillus*, *Actinobaculum*, Unclassified *Lactobacillales*, *Aerococcus*, *Porphyromonas*, *Gemella* ↓, *Neisseria* ↓, Unclassified *Clostridiales* ↑, *Campylobacter* ↑, *Haemophilus* ↓	/
Tractenberg et al.	Evaluate the effectiveness of intravesical probiotics instillation in alleviating urinary symptoms in NB patients	96 adults and 7 children with NB	Intravesical instillation	Culturelle (*L. rhamnosus* GG, 20 billion live organisms, instill once or twice within 30 hours after the onset of symptoms such as "cloudy" or "foul-smelling" urine, 6 month)	*Aerococcus* ↓, *Enterococcus*, *Escherichia*/*Shigella* ↓, *Klebsiella*, *Lactobacillus*, *Proteus*, *Pluralibacter*, *Streptococcus*, *Gardnerella*, *Veillonella*	/

UTIs, urinary tract infections; rUTIs, recurrent urinary tract infections; NB, neurogenic bladder; SCI, spinal cord injury; MRGN, multidrug-resistant Gram-negative bacteria; qd, once a day; bid, twice a day; qw, once a week.

#### 8.2.1 Probiotic therapy

*Lactobacillus* is considered a key microbial component of a healthy microbiome, and urinary studies in NB patients have revealed a marked decline in its abundance. As one of the most promising measures for UTIs prevention and treatment, *Lactobacillus* can modulate immune responses, inhibit the growth of pathogenic organisms, and reduce their adhesion to bladder epithelial cells (de Llano et al., 2017; Putonti et al., 2020; Tkhruni et al., 2020). For instance, *Lactobacillus rhamnosus* GR-1 has been shown to secrete various immunomodulatory proteins (such as GroEL, Elongation factor Tu, and NLP/P60), enhance NF-κB activity in T24 bladder cells (Petrova et al., 2021). It can downregulate the virulence of uropathogenic *Escherichia coli* and inhibit biofilm formation by pathogens via lectin-like proteins 1 and 2 (Petrova et al., 2016). Research has increasingly focused on using probiotics—particularly those based on *Lactobacillus* strains—to prevent UTIs and reduce their severity. However, *Lactobacillus* exhibits strain specificity, meaning that different strains vary in inhibitory mechanisms and effectiveness. This may require selecting specific probiotics or using combinations to target different pathogenic strains. The European Association of Urology guidelines recommend *Lactobacillus rhamnosus* GR-1, *Lactobacillus reuteri* B-54, *Lactobacillus reuteri* RC-14, *Lactobacillus casei strain Shirota*, and *Lactobacillus crispatus* CTV-05 for the prevention of rUTIs caused by various factors (Chen et al., 2023).

##### 8.2.1.1 Oral probiotics

In addition to regulating the inflammatory response, *Lactobacillus* also possesses the ability to migrate from the gastrointestinal tract through the rectum and anus to the urethra (Reid and Bruce, 2006). Early studies evaluated the oral probiotics approach positively, suggesting it could effectively prevent rUTIs (Falagas et al., 2006). However, recent research has indicated that the effectiveness of oral probiotics in preventing UTIs does not show satisfactory differences compared to antibiotics or placebo.

Beerepoot conducted a randomized, double-blind trial (ISRCTN50717094) comparing *Lactobacillus rhamnosus* GR-1 and *Lactobacillus* RC-14 with trimethoprim-sulfamethoxazole in 252 postmenopausal women suffering from rUTIs (Beerepoot et al., 2012). The administration of oral probiotic capsules significantly reduced the mean number of symptomatic UTIs among these women after 12 months, decreasing from 6.8 to 3.3. However, this did not satisfy the noninferiority criteria when compared to the antibiotic group, which also showed a decrease from 7.0 to 3.3. Another finding was that the probiotic group did not show an increase in bacterial resistance. However, the antibiotic group experienced an increase in resistance rates from 20%-40% to 80%-95%, highlighting the potential benefit of probiotics in avoiding antibiotic resistance. Wolff used the same oral probiotics method to investigate 7 healthy premenopausal women (NCT03250208) and found no significant change in the ratio of uropathogens to *Lactobacillus* in urine compared to a placebo (Wolff et al., 2019). Probiotics were not detected in the urine samples either. However, the study’s small sample size and reliance on EQUC for bacterial identification rather than high-throughput sequencing limit the conclusiveness of these findings.

NB patient studies have yielded comparable conclusions. A randomized, placebo-controlled trial (ACTRN12610000512022) conducted by Toh, utilizing identical oral probiotics in 207 SCI patients with NB, also demonstrated no significant effect of probiotics on preventing UTIs in this population (Toh et al., 2019). Nevertheless, *Lactobacillus rhamnosus* GR-1 and *Lactobacillus* RC-14 positively inhibited the colonization of multidrug-resistant Gram-negative bacteria (Toh et al., 2020).

In studies involving other probiotic combinations, Bossa observed that oral *Lactobacillus rhamnosus* GG with *Bifidobacterium* BB-12 could alter the catheter microbiome of SCI patients with NB during treatment (ACTRN12610000512022). However, this alteration is transient, the microbiome quickly returns to its original state after discontinuing the treatment (Bossa et al., 2017). Daniel conducted a study (NCT03462160) involving the oral administration of *Lactobacillus rhamnosus* PL1 and *Lactobacillus plantarum* PM1 in 54 children diagnosed with rUTIs, comprising 42 individuals with bladder dysfunction and 4 with NB (Daniel et al., 2020). The probiotic group showed a reduction in UTIs frequency and antibiotic treatment days, but these differences were not statistically significant compared to the placebo group.

Although oral probiotic therapy does not show a significant advantage over antibiotics in preventing UTIs, it mitigates the heightened risk of antibiotic resistance, indicating that probiotics may be a viable alternative to antibiotics. One plausible explanation for the comparable reduction in UTIs incidence observed in the placebo group is that participants may have unconsciously enhanced their aseptic techniques during clean catheterization and perineal care following enrollment, which is closely related to UTIs occurrences, though these relevant factors were not addressed in the studies. A recent study indicates that combining oral probiotic capsules ProBalans (containing *Lactobacillus acidophilus* LA3, *Bifidobacterium animalis* subsp. lactis BLC1, and *Lactobacillus casei* BGP93) with antibiotics provides better outcomes for UTIs treatment compared to antibiotics alone (Mula et al., 2024).

##### 8.2.1.2 Vaginal probiotics

The female urogenital microbiome exhibits interrelatedness, with samples from the same female urinary tract and vaginal revealing identical strains between these anatomical sites. This association extends beyond common pathogens, such as *Escherichia coli* and *Streptococcus*, to include beneficial bacteria closely linked to health, including *Lactobacillus iners*, *Lactobacillus crispatus*, *Lactobacillus jensenii*, and *Lactobacillus gasseri* (Thomas-White et al., 2018; Komesu et al., 2020; Atkins et al., 2024). A plausible hypothesis is that bacteria may transfer between the vagina and the urethra by “hitchhiking” with the movement of other microorganisms or through mechanical motion of adjacent areas (Muok and Briegel, 2021). This finding provides further theoretical support for the potential of influencing urinary microbiota through interventions targeting the vaginal microbiome.

The healthy vaginal microbiome is dominated primarily by *Lactobacillus*, which helps resist pathogen colonization by lowering vaginal pH and modulating immune responses (Holm et al., 2023). Some vaginal microbiome acting as a “hidden pathogen” that facilitates the pathogenesis of harmful bacteria, like *Gardnerella vaginalis*, can induce the release of *Escherichia coli* from latent bladder reservoirs leading to UTIs, trigger apoptosis and desquamation of bladder epithelial cells and mediate acute kidney injury through IL-1 receptor pathways (Lewis and Gilbert, 2020). Low abundance of *Lactobacillus* has been associated with higher disease risk, and supplementing with vaginal probiotics may effectively modulate the microbiome to prevent disease occurrence (Holdcroft et al., 2023).

Stapleton conducted a Phase II clinical trial (NCT00305227) involving vaginal suppositories containing *Lactobacillus crispatus* in 100 young women with a history of rUTIs (Stapleton et al., 2011). High levels of *Lactobacillus crispatus* colonization (≥10^6^ 16S RNA gene per swab from the vagina) were observed in 93% of the participants within the experimental group, which was associated with a significant reduction in UTIs incidence. In contrast, the placebo group demonstrated a *Lactobacillus crispatus* high-level colonization rate of only 68%, and there was no observed decrease in UTIs rates regardless of colonization levels, indicating that exogenous probiotic intervention provides a notable advantage over endogenous vaginal microbiome recolonization. Similarly, Sadahira utilized vaginal suppositories containing *Lactobacillus crispatus* to reduce the frequency of recurrent cystitis in postmenopausal women and confirm the successful colonization of lactobacilli in the vagina (Sadahira et al., 2021; Sekito et al., 2023).

A recent study shows that the combined use of vaginal probiotic suppositories with oral probiotics (jRCTs061180053) can significantly reduce the incidence of UTIs and alleviates patients’ discomfort (Gupta et al., 2024). However, current studies on transvaginal probiotic administration predominantly involve premenopausal or postmenopausal women, with limited research available on patients with NB and no relevant studies addressing changes in the urinary microbiome after treatment.

##### 8.2.1.3 Bladder instillation probiotics

It is difficult for bacteria to colonize the bladder, and successful bladder colonization partially depends on impaired bladder emptying (Wullt et al., 1998). The urodynamic defects observed in NB patients facilitate the establishment of non-virulent strains in the urinary tract.

Forster conducted a study about bladder instillation of *Lactobacillus rhamnosus* GG in 10 NB patients (Forster et al., 2021). They used standard urine cultures and 16S rRNA GS for multiple analyses of urine samples 7-10 days after the instillation. They discovered a significant disparity in *β* diversity of the microbiome before and after the instillation but no variance in the *α* diversity. After the instillation, significant shifts were observed in the relative abundances of *Escherichia*, *Prevotella*, and *Lactobacillus* in the urinary microbiome of most patients. However, the high individual heterogeneity made identifying consistent trends in specific taxa. Another study involving 103 adults and children with neurogenic lower urinary tract dysfunction due to SCI or other conditions described trends in urinary microbiome changes (Tractenberg et al., 2021; Groah et al., 2023). Participants used CIC to empty their bladders and performed bladder instillation one to two times when symptoms of foul-smelling or cloudy urine appeared. The study found that bladder instillation of *Lactobacillus rhamnosus* GG significantly reduced the α diversity of the urinary microbiome, along with a decrease in the abundance of *Escherichia*, *Shigella*, and *Aerococcus*. In contrast, there were no significant changes in the abundance of other urinary pathogens (such as *Klebsiella* and *Proteus*) or commensal bacteria (*Lactobacillus*, *Veillonella*, *Staphylococcus*, and *Streptococcus*).

Contrary to expectations, existing studies have not observed a consistent increase in *Lactobacillus* abundance following bladder instillation. The changes in the urinary microbiome may be attributed to the fluid’s mechanical flushing effect. Further efficacy studies are needed to confirm the role of probiotics. Currently, a randomized controlled comparative effectiveness clinical trial is underway, aiming to compare bladder instillation of probiotics with saline bladder irrigation (Groah and Tractenberg, 2022).

#### 8.2.2 Fecal microbiota transplantation

Fecal microbiota transplantation (FMT) aims to eliminate the colonization of pathogenic multidrug-resistant organisms in the gut, improve dysbiotic microenvironment conditions, and enhance resistance to pathogen colonization by transferring the fecal microbiota from healthy individuals to the gastrointestinal tract of specific patients through oral capsules or nasogastric tubes (Yoon et al., 2019; Wuethrich et al., 2021). FMT has successfully treated recurrent *Clostridium difficile* infections and reduced antibiotic-resistance genes associated with multidrug-resistant organisms (Nooij et al., 2024; Rashidi et al., 2024).

Due to the anatomical proximity of the urethra to the anus, the urinary system is often directly influenced by the microbiota of the digestive tract. Concurrently, the recent discovery of the brain-gut-bladder axis offers new insights into the mechanisms of UTIs (Worby et al., 2022). Dysbiosis of the gut microbiota has been linked to increased rUTIs, suggesting novel strategies for mitigating UTIs risk by enhancing the gut microbial environment (Choi et al., 2024; Ruţa et al., 2024).

The application of FMT in patients with UTIs caused by multidrug-resistant organisms has demonstrated promising clinical efficacy and economic benefits (Grosen et al., 2019; Baek et al., 2023). Hocquart conducted a study on patients suffering from both irritable bowel syndrome and rUTIs, 30g of feces from a healthy individual dissolved in 400 milliliters of saline and removed impurities, the solution was delivered via a nasogastric tube following an enema. During an 8-month follow-up, patients showed significant improvement in irritable bowel symptoms and no further occurrences of UTIs (Hocquart et al., 2019). Additionally, a case report by Bier highlighted the successful treatment of UTIs caused by extended-spectrumβ-lactamase -producing *Klebsiella pneumoniae* infection using an oral freeze-dried fecal capsule preparation known as PRIM-DJ2727 (60 grams of total stool lyophilized into 1 gram per dose), sequencing revealed an increase in urinary microbiome *α* diversity after FMT, accompanied by a reduction in the abundance of *Klebsiella pneumoniae* (Bier et al., 2023).

The role of FMT in the successful treatment of rUTIs may be complex. All studies have small sample sizes and lack systematic descriptions of dynamic changes in the urinary microbiome following FMT. Current research on FMT primarily focuses on gastrointestinal diseases (Porcari et al., 2023). In addition, addressing the potential psychological resistance patients may have toward fecal matter poses a challenge for future research (Yu et al., 2023).

#### 8.2.3 Phage therapy

Bacteriophages have shown high lytic activity against common uropathogens *in vitro* (Sybesma et al., 2016). Their movement and bacterial lysis efficiency are severely limited in low-humidity environments, making the liquid environment of the urinary system an ideal setting for medical applications (Żaczek et al., 2020). However, single phage therapy is limited in its ability to treat a variety of microbial infections due to its high specificity, and there is a risk of bacterial resistance to phages evolving. Hence, the combination of phages, phage lytic enzymes, and antibiotics is the preferred treatment approach (Malik et al., 2020).

Leitner conducted a randomized, double-blind, controlled trial (NCT03140085) involving 113 adult males with UTIs undergoing transurethral resection of the prostate. Participants received a 7 day bladder instillation of Pyophage (a commercial bacteriophage produced by the Eliava Institute) (Leitner et al., 2021b). The results showed no statistically significant differences in treatment efficacy among the groups (success rates: bacteriophage 18% vs. antibiotic 35% vs. placebo 28%). However, the bacteriophage treatment appeared to be more reliable regarding safety, with adverse event rates of 21% for bacteriophage, 30% for antibiotics, and 41% for placebo. Leitner also pointed out the potential use of phage therapy in NB individuals resulting from SCI and initiated a further clinical trial in 2020 to investigate phage therapy for catheter-associated UTIs (Leitner et al., 2021a).

The combination of bacteriophages and probiotics offers a promising new approach. Competitive alternative therapies using probiotics alone may be limited by ecological microenvironmental disorders in different sites such as the gut and vagina. Even if probiotics work well *in vitro*, a healthy gut or pathological bladder consists of already populated bacterial ecological niches, which may be at a disadvantage in the frequency-dependent competition for microenvironments when probiotics are administered orally or by bladder drip, which is in line with the widespread conclusion that probiotics have inconsistent clinical efficacy (Lerner et al., 2019). The efficient infection characteristics of bacteriophages may create favorable conditions for probiotics to colonize vacant niches, subsequently inhibiting or replacing target microorganisms through frequency-dependent competition (Forsyth et al., 2023). *In vitro* and animal studies have demonstrated superior synergistic effects of ST131-specific phages combined with *Escherichia coli* Nissle 1917, highlighting their potential clinical application for more effectively reducing or displacing uropathogens or combating the colonization of multidrug-resistant bacteria (Porter et al., 2022; Forsyth et al., 2023).

## 9 Conclusion

The study of the urinary microbiome in neurogenic bladder is gradually breaking through the conventional paradigm of sterile urine, revealing the complex ecology of the urinary system disrupted by neurological control mechanisms. It uncovers the cascade imbalance pattern in NB patients: “protective microbiota decline—pathogenic bacteria expansion—frequent UTIs—renal function damage.” Additionally, it identifies the synergistic effect of the disease’s natural progression and catheter insertion as an important intervention, providing evidence for defining clinical intervention windows.

Studies have confirmed that specific bacterial genera, such as *Lactobacillus* and *Enterococcus*, may be microbiological biomarkers, breaking through the diagnostic bottleneck. Constructing a UTIs risk stratification model based on microbial community characteristics provides auxiliary diagnostic tools. It promotes transforming the diagnostic and treatment model from “symptom-driven” to “microbial alert.”

More notably, microbiome-based therapies show potential in overcoming the dilemma of multiple drug resistance. By tailoring individualized treatment plans based on patients’ urinary microbiome baseline characteristics, a shift from passive infection control to active ecological regulation can be achieved.

However, there is limited research on NB, and some results exhibit heterogeneity. This heterogeneity may be attributed to small patient sample sizes, low urinary microbiome biomass, individual differences, variations in sample collection methods, and contamination during the collection process. Moreover, current research does not consider the different types of NB, and variations in urodynamics could result in individual differences in the urinary microbiome. Future research should refine methods to prevent the distortion of valuable results already obtained due to the aforementioned factors. Establishing standardized sampling protocols, reporting guidelines, and efficacy evaluation systems, along with developing a dynamic temporal database for the urinary microbiome in NB patients, will be essential. Furthermore, constructing a spatiotemporal microbiota-host interaction network that integrates the gut, vaginal, and urinary microbiomes following interventions such as probiotic therapy could provide deeper insights beyond those offered by cross-sectional studies.

In summary, current research on the urinary microbiome is facilitating the translation of basic research into clinical applications. The goal is to transform the urinary microbiome from an observational marker into a modifiable therapeutic target, reshaping the management paradigm of NB—from fragmented symptom control to precise ecological regulation, ultimately improving long-term patient outcomes and quality of life.
